# Editorial

**Published:** 1892-03

**Authors:** 


### Editorial.

### THE PROPER SPHERE OF SURGERY.

While operative measures upon any part of the organism come within the legitimate field of surgery, there are prerequisites in the recognition of the conditions warranting an operation, and in the preparation of the patient for undergoing it, which should characterize the true surgeon. Thus it may turn out that one who is habil, and dexterous in the use of instruments, is not properly qualified for the highest achievements in surgery, from lack of precaution.

The aim of the surgeon is a proper comprehension of the abnormality in all its bearings, and his end should be to afford relief with the least possible impairment of the functions of the part involved.

Meddlesome surgery is the outgrowth of ignorance and inexperience, while masterly inactivity may result from overcaution against doing harm; but the properly qualified surgeon will consider every phase of the case, and assume the responsibility of action or inaction regardless of the criticism of others.

Surgical pathology should not be limited to the macroscopic observation of the changes incident to inflammatory processes, but should include those modifications impressed upon the tissues by neurotic disorders, whether resulting in impairment or undue activity.

Surgical diagnosis should not be confined to what can be seen and felt, nor should surgical practice be circumscribed by the sphere of the knife or the ligature.

While the distinction of internal and external treatment is held by most European authorities to indicate the practice of medicine and surgery, it is not applicable in the broad sense of relegating all the disorders of the inner structures to the practitioner of medicine. Nor should we conclude that all the pathological conditions not calling for operative measures are beyond the domain of surgery. A more fitting basis of recognition for the different spheres of medicine and surgery would be that of functional and organic diseases. With this clear and definite limitation of the province of the physician, as contradistinguished from the field of the surgeon, the work of the former would be confined to such measures as are calculated to correct the functions of the different organs, whether internal or external.

Whenever organic changes ensue, the case then comes within the domain of surgery, whether it demands the use of medicine or a resort to operative measures. It presents conditions of structural modification, more or less developed, which alter the normal constituents so as to produce a change in the size, shape or density of the part involved. It may necessitate an incision or a puncture to diagnose such a state, which, upon being verified, would not warrant a radical operation; but if this change of structure could be clearly ascertained witho t an exploratory operation, it would none the less belong to surgery. With the line drawn between functional and organic disorders for the practitioner of medicine and surgery, so soon as a transition may be observed by the former, the case should be turned over to the latter.

Here lies the practical importance of the distinction submitted for due consideration by all concerned, and if a proper discrimination is made by the physician in the course of his treatment, there will not be so many instances of surgeons being called on to lead a forlorn hope in battling with the latter stages of disorders.

The want of proper investigation of all the symptoms in a case presenting grave complications may induce a physician to delay calling upon a surgeon until the opportunity for relief has passed and his interference would avail nothing.

A timely consultation may arrest such an undesirable result and is earnestly urged upon practitioners J. McF. G

---

I have used Peacock's Bromides with marked success in cases of hysteria, cerebral congestion, convulsions, spinal meningitis, and in fact all nervous diseases.

John Croft, M. D., Detroit. Mich.

### THIRTEENTH COMMENCEMENT OF THE SOUTHERN MEDICAL COLLEGE.

The Southern Medical College held its thirteenth annual commencement Thursday afternoon, March 3d, at DeGive's opera house.

During the term ninety-five students were in attendance at the lectures, forty-six of whom were seniors and forty-nine juniors, representing almost all of the Southern States and many of the Northern.

Of the forty-six senior students, thirty-four were presented as entitled to the degree of doctor of medicine. The graduates were as follows: A. Mayfield Allen, South Carolina; William A. Allen, Georgia; Peter M. Baker, Alabama; Thomas B. Bonner, Georgia; William W. Bonner, Alabama; George Brown, Georgia; William J. Cox, Georgia; Alton R. Danforth, Georgia; John H. Garrett, Alabama; Charles J. George, Georgia; Herbert L. Gill, Georgia; William R. Groover, Florida; William T. Friddell, Georgia; Grant P. Fuller, New York; John D. Hall, Alabama; Lewis M. Hobgood, Georgia; Robert M. Hood, Georgia; Morgan M. Howle, Alabama; John W. Jinks, Georgia; William P. Knight, Alabama; D. Pugh Miller, Gecrgia; William A. Moore, Georgia; Fredrick M. Mullino, Georgia; Calvins Parker, Georgia; John E. Prichard, Georgia; William A. Ross, South Carolina; Christopher R. Rushton, Alabama; W. Marshall Shepherd, Georgia; James R. Smith, Georgia; Reuben C. Stevens, Alabama; Charles L. Tucker, Georgia; Joseph J. Waller, Tennessee; James M. Welch, Alabama; Beauregard Williams, Georgia.

Dr. Joseph J Waller, of Tennessee, took first honor. He is the first man that has ever made a ninety-five out of a possible ninety-six. The first prize was a gold medal.

The second honor man was Dr. W. J. Cox, of Georgia. The second prize was an amputating case.

Dr. Thomas B. Bonner, of Georgia, took third honor, and was awarded a case of pocket instruments.

The following gentlemen were granted honorable mention: Dr. William R. Groover, of Florida; Dr. James M. Welch, of

Alabama; Dr. Christopher R. Rushton, of Alabama; Dr. A. Mayfield Allen, of South Carolina.

After the diplomas had been delivered, Dr. Powell, president of the college, made an able address to the young men which was highly entertaining.

Dr. George Brown, of Georgia, was the valedictorian for the class, and his speech was one that his classmates and those who were fortunate enough to hear, will long remember.

The exercises were closed with an oration by the Rev. Dr. Robbins, annual orator for the occasion.

Dr. William Perrin Nicolson, the dean, gave over the medals, prizes and presents with hearty words of cheer to the class.

The annual banquet was held at Vignaux's, at 8:30 o'clock the same evening, and was a complete success.

Dr. George Brown acted as toastmaster, and in the happiest manner imaginable discharged his duties.

Dr. T. S. Powell, president of the college, responded to the first toast. It was "The New College." Dr. Powell's response was a decided hit.

Dr. G. G. Roy responded to "Therapeutics—Ancient and Modern," and did it in a most captivating style.

Dr. J. McFadden Gaston discoursed upon "Conservatism with Discretion—the Highest Attribute of the Surgeon," and was discreet in his conservative sayings.

"Medical Education" gave the dean, Dr. William Perrin Nicolson, a chance to tell those present many good things.

Dr. W. S. Elkin responded to the toast of "Surgery, Past and Present.

Dr. Charles S. Webb spoke on "My New Diploma."

Dr. C. D. Roy and Dr. L. B. Grandy responded happily to "The Next Generation in Medicine."

"The Dental Department," was Dr. Sid Holland's theme.

"The Law Department," was most happily responded to by Mr. Charles A. Read.

Dr. J. W. Price gave a response to "The Alumni Association of the Southern Medical College."

The evening was closed by the organization of an Alumni Association, with the following officers: Dr. John W. Price, President; Dr. George T. Brown, Vice-President, and Dr. C. Evans Johnson, Secretary and Treasurer.

### THIRTY-FOURTH COMMENCEMENT OF THE ATLANTA MEDICAL COLLEGE.

The graduating exercises took place at DeGive's Opera House, and a very large and brilliant audience were present.

The exercises were opened at 8 o'clock with prayer by Rev. James W. Pogue, immediately after which Dr. W. S. Kendrick, proctor of the college, made his report.

The first year's work of the College of Pharmacy was gratifying, and the success has been even more than anticipated.

The number of students during the year was 181, distributed as follows:

From Georgia, 132; Alabama, 11; South Carolina, 8; North Carolina, 6; Texas, 4; Florida, 3; Louisiana, 2; New York, 2; Tennessee, 1; Mississippi, 1; Ohio, 1.

The report showed that this was the largest class in the history of the college. All were pleased with the showing.

Hon. N. J. Hammond, president of the board of trustees, delivered the degrees.

Following is a list of the graduates:

V. H. Taliaferro, Atlanta, first honor; H. W. Terrell, Green ville, Georgia, second honor; J. H. Latimer, Hazlehurst, Georgia, third honor. Honorable mention of Alfred F. Harrington, West Point; T. V. Hubbard, J. F. Yarborough, W. A. Sheldon, S. W. Hart, with the following graduates: M. P. Anthony, H. M. Barton, A. H. Baskin, R. S. Bryan, G. B. Carter J. A. S. Chambers, A. J. Cooper, R. T. Crozier, J. J. Crumbly, Everett Daniel, D. C. Daves, T. N. Dunn, A. L. Franklin, H. A. Gantt, G. M. Gold, J. B. Goldin, W. S. Goldsmith, R. L. Goodbred, J. R. Hall, J. C. Hardy, A. F. Harrington, W. S. Hill, J. W. Hinton, Jr., C. W. Holloway, R. L. Hughes, A. S. Humphreys, J. A. Hutson, J. F. Johnson, W. E. Johnson, Robert Jones, J. A. Kitchens, Lucius Lamar, F. L. Lewis, E. P. Little, R. C. McKown, Thomas McLeod, W. H. Mann, F. J. Mashburn, W. F. May, J. H. Morgan, M. W. Murphy, C. T. Nolan, J. B. Owens, S. B. Patton, G. F. Payne, A. A. Powell, J. F. Reid, E. C. Ripley, W. C. Sessions, W. A. Shelman, C. O. Smith, S. S. Smith, F. V. Turk, W. P. Turk, A. R. Watkins, G. R. Wells, F. L. Wood, D. A. York, J. H. Barr.

Judge Howard Van Epps was orator of the occasion, and made a good speech that was well received.

Dr. J. A. S. Chambers, the valedictorian, made a charming parting address, and received much applause.

Hon. E. W. Martin gracefully delivered the medals to the three honor men, and the exercises were closed.

---

### THE MEDICAL ASSOCIATION OF GEORGIA.

Meets April 20th, 1892.

The approaching meeting of our State Association in Columbus should interest all the profession, and it is hoped that there may be a full representation from the different sections of the State. Many advantages accrue from these annual re-unions of practitioners, and each should contribute his part towards the general fund of information, either by preparing a paper, giving practical observations, or joining in the discussion of subjects at the meeting. But should any one not care to join in the work of writing or discussion, an important service may still be rendered by his presence, to encourage others in performing their duties, and a good listener will reap much profit by attendance. It should not, however, be supposed that a member of the profession who is not a ready writer, or a fluent speaker, is not fitted to present a useful report of his cases, and frequently the observations of this class of practitioners prove highly instructive to his colleagues. Let no one cover his light under a bushel, but come forth with his statement of facts, as viewed from his individual standpoint, thus lending a helping hand in the mutual benefits of comparing the results of experience in the treatment of diseases. There is no preferred class in the medical profession, and all being on an equal footing, each should exercises his right and privilege to present his opinions, so that the truth may prevail in reaching our conclusions.

While the program committee is charged with getting up papers and arranging for their discussion at the meeting, this does not preclude voluntary contributions, and even at the last moment, a report of some instructive case may be made by some one who has not found time to get a paper ready previously. Every member of the medical profession should take pride in promoting the end of the Association, and attend this meeting. J. McF. G.

### A DELIGHTFUL RECEPTION.

One of the most charming receptions ever given in Atlanta, was that to the graduating class of the Southern Medical College, given last night at the residence of Dr. and Mrs. G. G. Roy.

The full faculty, each professor with his lady, was present, and a number of Atlanta's most popular young ladies graced the occasion and added infinite pleasure to the happy young physicians.

Almost one hundred guests were present, and a more pleasant evening was never spent.

Dr. and Mrs. Roy are hospitality itself, and made all feel perfectly at home, in their elegant home. The refreshments were delightful, and the new doctors will never forget the closing evening.

### MEDICAL ASSOCIATION OF GEORGIA.

The forty-third annual session of the Medical Association of Georgia will meet in Columbus, Ga., on April 20, 21, 22.

The officers are: President, G W. Mulligan, M. D., of Wasington, Ga.; Vice Presidents, James M. Hull, M. D., of Augusta; Mark H. O'Daniel, M. D., of Macon; Treasurer, E. C. Goodrich, M. D., of Augusta; Secretary, Dan H. Howell, M. D., of Atlanta. DAN H. HOWELL, M. D.

Secretary Medical Association of Georgia.

DEATH OF DR. CARPENTER. Dr. Alfred Carpenter, of London, author of Carpenter's Human Physiology, died on Jan. 27th.

SIR WILLIAM THOMPSON has been raised to the peerage.

The new crematorium at Heidelberg was used for the first time on December 23d.

MR. BERKLY HILL, Vice-President of the Royal College of Surgeons, England, is dead.

